# Identification and validation of key mitophagy-related biomarkers in the pathogenesis of proliferative diabetic retinopathy

**DOI:** 10.3389/fendo.2025.1652898

**Published:** 2025-10-29

**Authors:** Yifan Liu, Ye Zhou, Jie Chen, Jiahui Jin, Xueying Wang, Xi Wang, Jieping Zhang, Jiao Li, Junfang Zhang, Ling Zhu, Guo-Tong Xu, Yanlong Bi, Qingjian Ou, Caixia Jin

**Affiliations:** ^1^ Department of Ophthalmology and Laboratory of Clinical and Visual Sciences of Tongji Eye Institute, Tongji Hospital, School of Medicine, Tongji University, Shanghai, China; ^2^ Life Sciences Experimental Teaching Center, School of Medicine, Tongji University, Shanghai, China; ^3^ Stem Cell Research Center, School of Medicine, Tongji University, Shanghai, China

**Keywords:** proliferative diabetic retinopathy, mitophagy, differentially expressed genes, CASP8, COL1A1

## Abstract

**Introduction:**

Diabetic retinopathy (DR) is one of the most common microvascular complications of diabetes mellitus, and proliferative diabetic retinopathy (PDR) represents its advanced stage. The etiology of PDR is complex. Mitophagy, the selective degradation of dysfunctional mitochondria, is crucial for cellular homeostasis and has been implicated in PDR pathogenesis. However, its specific mechanisms require further investigation.

**Materials and methods:**

Gene Expression Omnibu (GEO) datasets (GSE102485, GSE60436) were analyzed in R software to identify differentially expressed mitophagy-related genes (DEMRGs). A PDR diagnostic model was constructed by gene ontology (GO) enrichment analysis, genome enrichment analysis (GSEA), and other relevant methods. Immune infiltration was also performed to analyze the changes in immune cells. Finally, the retinal pigment epithelial cell line (ARPE-19) was incubated with high glucose (HG) to simulate a DR model *in vitro*, hub-gene expression and mitophagy were assessed by qRT-PCR, Western blotting, and immunofluorescence microscopy (IF).

**Results:**

Eight DEMRGs were identified enabling construction of a PDR diagnostic model and prioritization of two hub genes (CASP8 and COL1A1). Finally, qRT-PCR, Western blotting, and IF were performed to provide preliminary validation of the PDR model and HG stimulation increased mitochondria–lysosome colocalization as well as enhanced the expression of mitophagy-related proteins.

**Conclusion:**

Integrated bioinformatics and experimental validation suggest that mitophagy contributes to PDR pathogenesis. Five DEMRGs showed up-regulated and immune cell infiltration that may affect the occurrence and PDR development by regulating mitophagy. These findings provide candidate biomarkers and mechanistic insight into PDR.

## 1 Introduction

Diabetic retinopathy (DR) is one of the most common and detrimental microvascular complications of diabetes mellitus and a common eye disease that causes blindness (1). DR is estimated to affect 34.6% (93 million individuals) of the global population aged 40 (2). Proliferative diabetic retinopathy (PDR) is an advanced diabetic eye disease. Due to abnormal neovascularization on the surface of the retina or optic disc in patients with PDR, extensive retinal ischemia and hypoxia occur, leading to loss of central and peripheral vision, and approximately half of the untreated patients with proliferative retinopathy will be blind within 5 years (3). Consequently, PDR imposes substantial personal and societal burdens on patients, their families, and the healthcare system.

Chronic low-grade retinal inflammation is recognized as a central feature of DR pathophysiology. Converging evidence have shown that oxidative stress, mitochondrial damage (4), mitochondrial dysfunction (5), dysregulated apoptosis (6), and defects in autophagy disrupt the blood–retinal barrier (7), pathological angiogenesis, and neurodegeneration. Mitochondria orchestrate these stress responses, and their quality control is partially mediated by mitophagy, the selective autophagy of damaged mitochondria that is essential for maintaining a healthy mitochondrial network (8, 9). In humans with diabetes, mitochondrial status and mitophagy follow a temporal, dynamic progression. During early hyperglycemic stress, mitochondria face heightened energetic demand and oxidative burden, accompanied by oxidative damage to mitochondrial DNA. As a quality control response, mitophagy is engaged pre-apoptotic ally to cull compromised organelles. With persistent hyperglycemia and redox imbalance, however, clearance capacity lags, dysfunctional mitochondria accumulate, and structural and functional deterioration of the retinal neurovascular unit ensues. Accordingly, preserving mitochondrial homeostasis and enhancing mitophagic capacity may represent promising therapeutic avenues (10). When mitochondrial turnover is impaired, the release of cytochrome c, apoptosis-inducing factor (AIF), and endonuclease G promotes mitochondria dependent cell death and contribute to retinal disease (11, 12).

Mitophagy is increasingly implicated in ocular disorders. In age related macular degeneration (AMD), excessive reactive oxygen species (ROS) activate mitophagy in the retinal pigment epithelium (RPE) via the p62/Keap1/Nrf2 axis (13, 14). Recently, the role and multiple function of mitophagy in PDR has been revealed. In DR, RPE dysfunction activates mitophagy at low glucose levels and inhibits it at late high glucose (HG) levels, leading to decreased visual function (6). However, the mechanism of mitophagy in RPE cells under diabetes stress remains unclear.

This study analyzed a previously published dataset containing samples from PDR and non-diabetic individuals to identify the differentially expressed genes (DEGs) associated with PDR. The GSE102485 dataset includes samples obtained from the retina and optic disc of patients with PDR, whereas GSE60436 comprises fibrovascular membranes (FVMs) from patients with PDR and normal human retinal tissue. Further analysis was performed to determine the correlation between differentially expressed mitophagy-related genes (DEMRGs) in PDR. PDR pathogenesis was investigated using Gene Ontology (GO), Kyoto Encyclopedia of Genes and Genomes (KEGG) pathway enrichment and protein-protein interaction (PPI) analyses. Mitophagy-related genes and pathways were analyzed using the MSigDB database to further explore the pathogenesis, pathophysiology, and molecular mechanisms of PDR. Eight genes (VGF, SNX30, IFIH1, CASP8, UTRN, ITGA5, COL1A1, and MYH9) DEMRGs and two hub genes (CASP8 and COL1A1) were highlighted. Subsequently, we established high-glucose (HG) models in human retinal pigment epithelial cell line (ARPE-19) cells and in human induced retinal pigment epithelium (iRPE) cells derived from human umbilical cord mesenchymal stem cells (hUCMSCs) (15). HG treatment resulted in elevated mitochondria-lysosome colocalization and enhanced expression of mitophagy-related proteins in RPE cells. The PDR model was preliminarily validated at both the mRNA and protein levels. Consistency between the expression of SNX30, IFIH1, CASP8, UTRN, and COL1A1 in the PDR model and bioinformatic analyses, suggesting that these genes may be involved in PDR pathogenesis, potentially through modulation of mitophagy.

## 2 Materials and methods

### 2.1 Datasets acquisition

The GSE102485 sequencing and GSE60436 microarray datasets for patients with PDR and controls were obtained from the public GEO dataset (16). The GSE102485 (Homo sapiens) dataset encompasses 30 samples: 22 from patients with PDR, 3 from control patients, 3 from patients with branch retinal vein occlusion, and 2 from patients with peripapillary retinal peri phlebitis. The GSE60436 dataset was derived from a Human Whole Gene Expression Profiling GeneChip (Illumina HumanWG-6 v3.0). This data has 9 samples: 6 from patients with PDR and 3 from control patients.

The GSE102485 dataset uses raw read count data. After a counts per million (CPM) transformation by the edgeR package, 22 PDR samples and 3 control samples were included in a training set (Training) for downstream analysis, among the tissue sources for patients with PDR are the hemal arch, the nasal side, the entire portion, the retina, the hemi-arch portion, and the optic disc, the optic disk, and the peripheral portion of the optic disc (17). The GSE60436 dataset was normalized using the limma package and annotated with data from the GPL6884 microarray platform. The full sample was included as a validation set (Validation) for downstream analysis (18). The mitochondria were obtained by searching the MSigDB database (https://www.gsea-msigdb.org/gsea/msigdb/index.jsp) and the GeneCards database (https://www.genecards.org/) with the keywords mitophagy, autophagy-related genes, and pathways. Following de-emphasizing and merging, 2414 mitophagy-related genes (MRGs) were found. **Supplementary Table S1** provides comprehensive details (19, 20).

### 2.2 Immune infiltration analysis

xCell, an approach based on gene characteristics (21), we conducted a cell type enrichment analysis on the Training dataset and extracted information on 28 different immune cell types, including various subsets of B cells, T cells, memory cells, and other immune cell populations (**Supplementary Table S2**). We compared the immune enrichment levels in the PDR and control groups, mapped the immune infiltration distribution of various samples, and examined the association between the various immune cell enrichment levels.

### 2.3 Differentially expressed gene analysis

The DESeq2 package was applied to clarify the DEGs in patients with PDR relative to normal patients and to further infer the pathways and functions of the differences (22). The criteria |logFC|>1 and adj.Pvalue < 0.05 were used to filter and select DEGs with statistical significance. Subsequently, we visualized the results through the creation of volcano plots and heatmaps using the ggplot2 and pheatmap packages. PDR-associated DEMRGs were defined as the intersection of DEGs and MRGs; the overlap was visualized with Venn diagrams using the ggvenn package and used in subsequent analyses.

### 2.4 Enrichment analysis

Gene Set Enrichment Analysis (GSEA) was employed to evaluate disparities in biological processes between healthy individuals and those with PDR (23). The R package clusterProfiler (24) was employed to analyze the GO, KEGG, and DO of DEMRGs using the enrichment criteria of p-value < 0.05 and q-value < 0.05, as well as using Benjamini-Hochberg (BH) p-value correction. In addition, we obtained the c2.cp.v7.5.1.symbols.gmt gene set from the MSigDB database and analyzed the differential BP between the PDR and control groups using the clusterProfiler package with the random seed set to 123456, the screening criterion of p-value < 0.05, and the BH p-value correction method. To perform GSEA, we used the Gene Set Enrichment Analysis (GSVA) package for one-sample enrichment of the training dataset, with the nonparametric estimation method set to Gaussian, and analyzed the difference in pathway enrichment scores between the two groups using the Wilcoxon rank-sum test for different pathway enrichment scores. The differential pathway screening criteria were |logFC|>1 and adjusted p-value < 0.05, as demonstrated by heatmaps.

### 2.5 Constructing diagnostic models

Least Absolute Shrinkage and Selection Operator (LASSO) regression is a technique employed to reduce the dimensionality of data. The implementation of LASSO is facilitated through the glmnet package (25) with the family parameter configured as binomial and the random seed set to 123456. Random forest is an integrated machine learning method that is used to resolve the defects inherent in a single model or set of parametric models by integrating more models, thereby combining their strengths and weaknesses to mitigate limitations. For this study, the mtry parameter was set to 6, the ntree parameter was set to 500, and the random seed was set to 123456. We constructed a diagnostic model using two machine learning methods and used the area under the curve (AUC) to verify the accuracy of the model in both Training and Validation datasets. In addition, the feature genes screened by machine learning were selected to take the intersection as the model feature genes for PDR.

### 2.6 Weighted gene correlation network analysis

Weighted gene correlation network analysis (WGCNA) is a method rooted in systems biology that serves to elucidate and characterize patterns of gene associations across diverse samples (26). We used the expression matrix of the DEGs as the input file and used pickSoftThreshold to calculate the optimal soft threshold. We used these results to construct a scale-free network, calculate the topology matrix, and perform hierarchical clustering. The minimum number of module genes was set to 30 to construct gene modules; similar modules were merged by setting the minimum distance of the merged modules to 0.2. Correlation analysis was used to determine the correlation between each module and the clinical characteristics. The module with the highest correlation with PDR was selected as the core module.

### 2.7 Protein-protein interaction analysis

We leveraged the DEMRGs and the STRING (Search Tool for the Retrieval of Interacting Genes/Proteins) databases (https://cn.string-db.org/) to craft a PPI network (27). To create the PPI network diagram (**Supplementary Figure S1**), an interaction score of medium confidence, set at 0.4, was used. The Maximal Clique Centrality (MCC) (28) algorithm in Cytoscape (29) was used to find the core nodes in the PPI network. The top 50 MMC values were selected as candidate genes.

We amalgamated the outcomes stemming from the model feature genes, the central module identified through WGCNA, and the PPI network. By intersecting these sets of candidate genes, we derived a subset (the core, or hub, genes) that represents the central elements of interest in our analysis.

### 2.8 Identification of PDR molecular subtypes

The ConsensusClusterPlus package (30) an R package of choice, configures the clustering algorithm as partitioning around medoids (PAM) using the Pearson method for calculating distances and setting the number of re-samplings to 1000, the resampling ratio to 0.8, and the random seed to 123456. We utilized hub genes and their expression matrices in the consensus clustering of PDR samples and compared the correlation of hub genes in different subtypes with the enrichment of immune cells between different subtypes.

### 2.9 Cell culture

DMEM/F12 medium (Gibco, USA) supplemented with 10% heat-inactivated fetal bovine serum (FBS) (Gibco, USA), penicillin (100 U/ml), and streptomycin (100 µg/ml) was used to culture ARPE-19 and iRPE (15). Cells were cultured at 37°C in a humidified atmosphere with 5% CO2. The sub-culturing was performed by treating the cells with a 0.05% trypsin–EDTA solution (Gibco, USA). Cells in the logarithmic growth phase exhibiting good condition were seeded into 6-well plates at a density of 1.2 × 10^6^ cells per well. The cells were divided into two groups: a high glucose (HG) treatment group and a normal control (NC) group. The HG group was cultured in medium containing 30 mmol/L D-glucose, while the NC group was cultured in standard glucose (SG) medium. A volume of 2.5 mL of the respective medium was added to each well, and the cells were incubated at 37°C for 48 hours.

### 2.10 Quantitative real-time polymerase chain reaction

Total RNA was extracted and reverse transcription was performed using Primescript™ RT Master Mix kit (Takara, Shiga, Japan). qRT-PCR was performed in a Chromo4 instrument cycler (Bio-Rad, Hercules, USA) using Super-real Premix plus kit (Tiangen Biotech, Beijing, China). qRT-PCR amplification was carried out with the following cycling parameters: denaturation at 95°C for 5 min, followed by 40 cycles of 95°C for 30 s, 60°C for 30s. Primer sequences (Synthesized by Sangon Biotech Co., Ltd., Shanghai, China) were listed in **Table 1**.

**Table 1:** Primers and their sequences for qRT-PCR analysis.

Gene Symbol (Human)	Forward (5’-3’)	Reverse (5’-3’)
GAPDH	CAAGAGCACAAGAGGAAGAG	CTACATGGCAACTGTGAGG
VGF	GTGTGAAGTGTGTCTGTCTC	AACAGAGAAAGGAAAGAAGGG
SNX30	CTGTCATCTCGGCCTTTATC	GGAATCCACCAGACTTCATC
IFIH1	CACAGTGGTTCAGGAGTTATC	GCATACTCCTCTGGTTTCATATT
CASP8	CTTTGACCACGACCTTTGA	TGGTCCATGAGTTGGTAGA
UTRN	CCCAGATGGAAAGGACTAATG	GGCAATACTGCTGGATGAG
ITGA5	CACATCGCTCTCAACTTCTC	TCTGAGCCTTGTCCTCTATC
MYH9	GGACCTTCCACATCTTCTATTA	GGACAGGAAGCGGTATTT
COL1A1	CCTGTCTGCTTCCTGTAAACTC	GTTCAGTTTGGGTTGCTTGTC

### 2.11 Western blotting

The ARPE-19 cells were lysed by RIPA buffer containing protease and phosphatase inhibitor (C0001 and C0004, TargetMol, USA). The protein extracts (20 µg per sample) were separated by 10% SDS-PAGE gels and transferred onto polyvinylidene difluoride membranes (Millipore, Bedford, MA, USA). After being blocked with 5% BSA in TBST for one hour, membranes were incubated with primary antibodies against CASP8 (Abcam), COL1A1 (Abmart), LC3B (CST), P62(SQSTM1), PINK1, Parkin, ATP5A1and ACTB (Proteintech) for 12 hours at 4°C, followed by incubation with corresponding secondary antibodies for one hour at room temperature. The blots were visualized with a chemiluminescence imaging system (Tanon 5200; Tanon Shanghai, China) and quantified with Image J software (Version 1.48v). Antibodies for Western Blot were listed in **Supplementary Table S3**.

### 2.12 Autophagy detection through lysosome and mitochondria colocalization

The mitochondria of live ARPE-19 cells were stained with a working solution of Mito-Tracker Red (Beyotime, China) at a concentration of 100 nM and incubated at 37°C for 20 min as our previous report (14). Briefly, the cells were stained with a working solution of Lyso-Tracker Green (50nM, Beyotime, China) for 15 min. The samples were then examined by fluorescence microscope (Olympus IX73, Tokyo, Japan). Co-localization analysis of lysosomes and mitochondria was performed using ImageJ software.

### 2.13 JC-1 staining

Live ARPE-19 cells were stained with 1 mL of JC-1 working solution (Beyotime, China) and incubated at 37°C for 20 min. After incubation, the supernatant was aspirated and cells were washed twice with JC-1 staining buffer. JC-1 aggregates present in normal mitochondria show red fluorescence, while JC-1 monomers present in unhealthy mitochondria produce green fluorescence. The samples were then examined by fluorescence microscope (Olympus IX73, Tokyo, Japan) and quantitative analysis (aggregates/monomers fluorescence ratio) was performed using ImageJ software.

### 2.14 Statistical analysis

In this study, all data computations and statistical evaluations were conducted using R software (version 4.1.2). When comparing the two groups with continuous variables, we employed the Wilcoxon rank-sum test to assess differences, particularly in cases where the variables did not follow a normal distribution. The results were calculated using Pearson correlation analysis of the correlation coefficients between different molecules. A p-value < 0.05 was considered statistically significant.

## 3 Results

### 3.1 Data pre-processing

**Figure 1:**
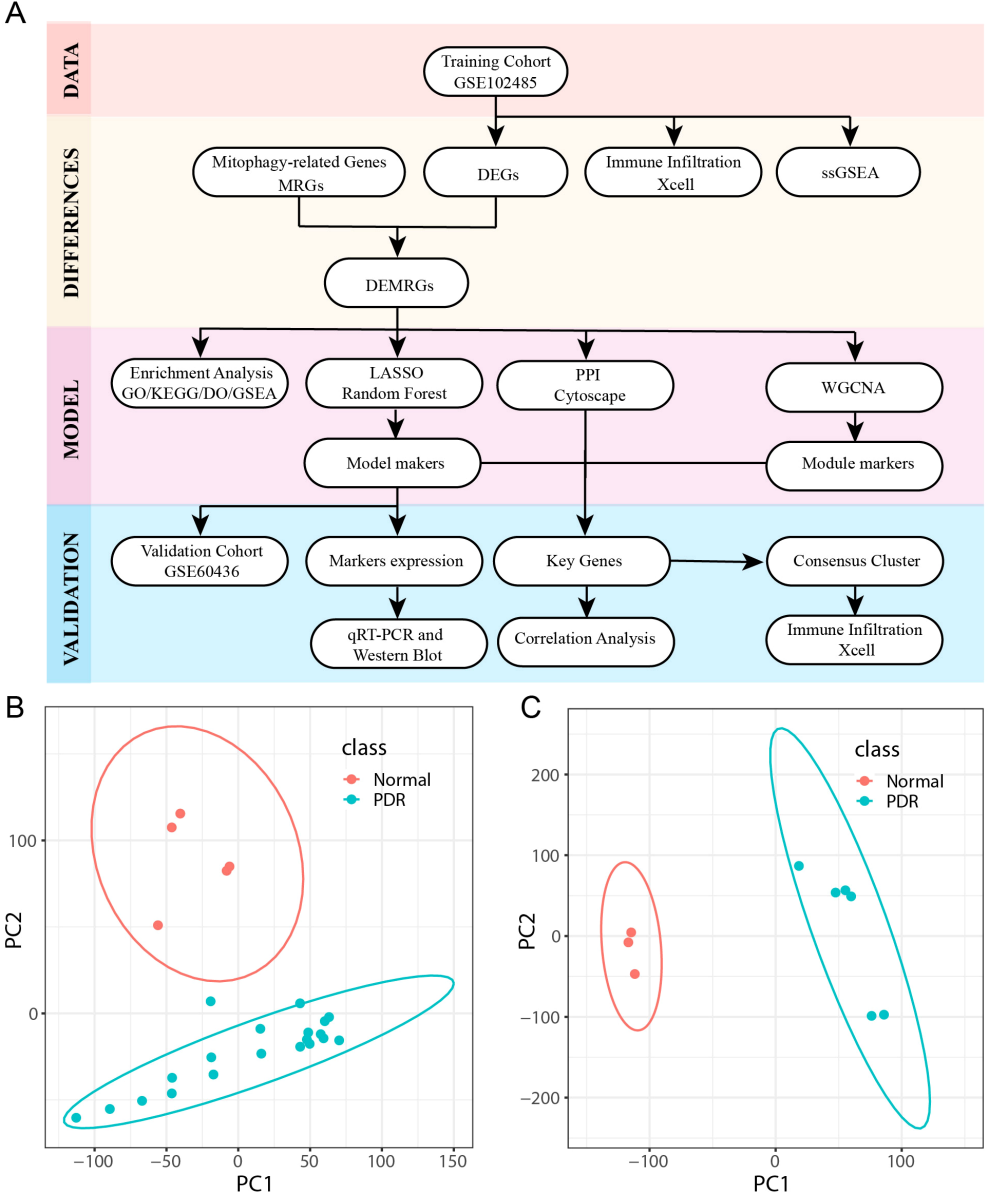
Flowchart and dataset. **(A)** Workflow for Identifying Mitophagy-Related Signatures in Proliferative diabetic retinopathy (PDR). **(B, C)** Principal Component Analysis (PCA) downscaling of GSE102485 and GSE604361 datasets (PDR in class denotes proliferative diabetic retinopathy samples and Normal represents normal control samples).

The study design and procedures are shown in **Figure 1A**. Detailed data regarding the datasets are presented in **Table 2**. Principal component analysis was used to effectively visualize the data distribution. Biological differences were found between PDR and control samples and good clustering was observed between samples of the same type (**Figures 1B, C**).

**Table 2:** Overview of the dataset.

GEO number	Date of publication	Type of organization	Volume of data	Chip platform
GSE60436	2014	FVMs from PDR (type II)vs normal human retinas	9	GPL6884
GSE102485	2020	NVM from PDR, BRVO and normal	30	GPL18573

### 3.2 Immune cell infiltration patterns and correlations

**Figure 2:**
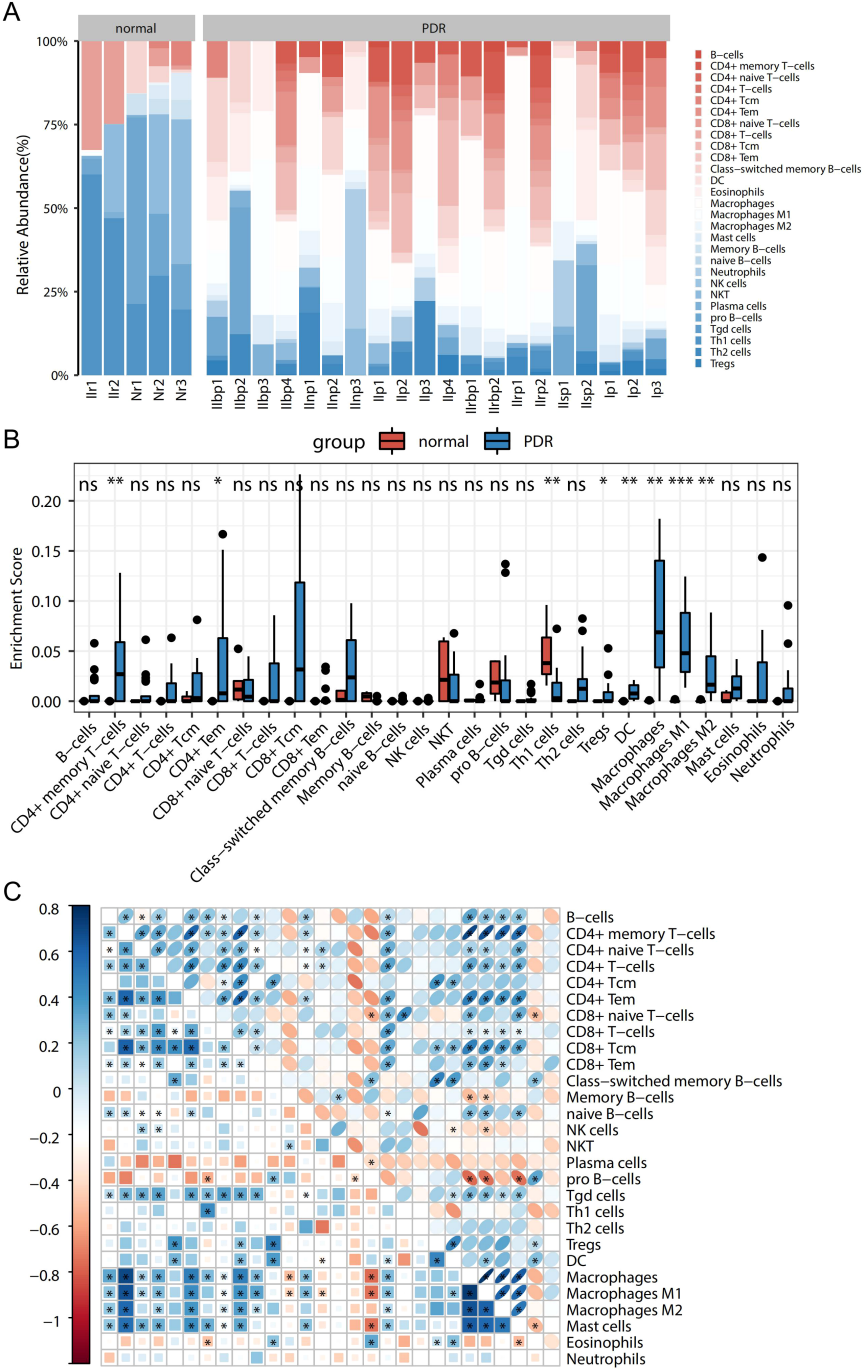
Panoramic view of disease immunocyte infiltration in the training dataset and correlation analysis. **(A)** Panoramic view of the infiltration of 28 immune cells between in the PDR and Control groups. **(B)** Difference of each immune cell between the PDR and Control groups (*p<0.05; **p<0.01; ***p<0.001). **(C)** Heatmap of the correlation between immune cells.

To explore the immune-related mechanisms in the PDR process, the enrichment scores of 28 immune cells in the Training dataset were analyzed to obtain a panorama of immune cell infiltration in each sample. This provided a comprehensive overview of immune cell infiltration in each sample (**Figure 2A**). The enrichment scores differed greatly in terms of immune cell infiltration between PDR and control samples. When comparing PDR samples to control samples, an increase was observed in the percentages of CD8-positive T lymphocytes (CD8+) and CD4-positive T lymphocytes (CD4+), dendritic cells (DCs), and macrophages, whereas a decrease was observed in the percentages of B cells, NK cells, and regulatory T cells.

Further exploration involved the comparison of immune cell differences among various sample types (**Figure 2B**). Notably, this study found significantly higher enrichment scores for CD4+ memory T cells, CD4+ macrophages, and their isoforms in the PDR group than in the control group. Conversely, the enrichment scores for Th1 cells were notably higher in control samples than in PDR samples. These are consistent with broader immune cell infiltration pattern.

Additionally, the correlations among the 28 immune cell types were studied, as presented in **Figure 2C**. Different immune cells exhibit varying degrees of correlation. Specifically, the highest positive correlation was discovered between macrophages and CD4+ memory T cells (with a correlation coefficient of 0.89), while the most significant negative correlation was observed between macrophages and pro-B cells (with a correlation coefficient of -0.56).

### 3.3 Identification of 540 differentially expressed mitophagy-related genes

Of the 3957 DEGs screened, 2460 genes were up-regulated and 1497 genes were down-regulated (**Supplementary Figure S2A**). The expression levels of DEGs were significantly different between the PDR samples and control samples (**Supplementary Figure S2B**).

**Figure 3:**
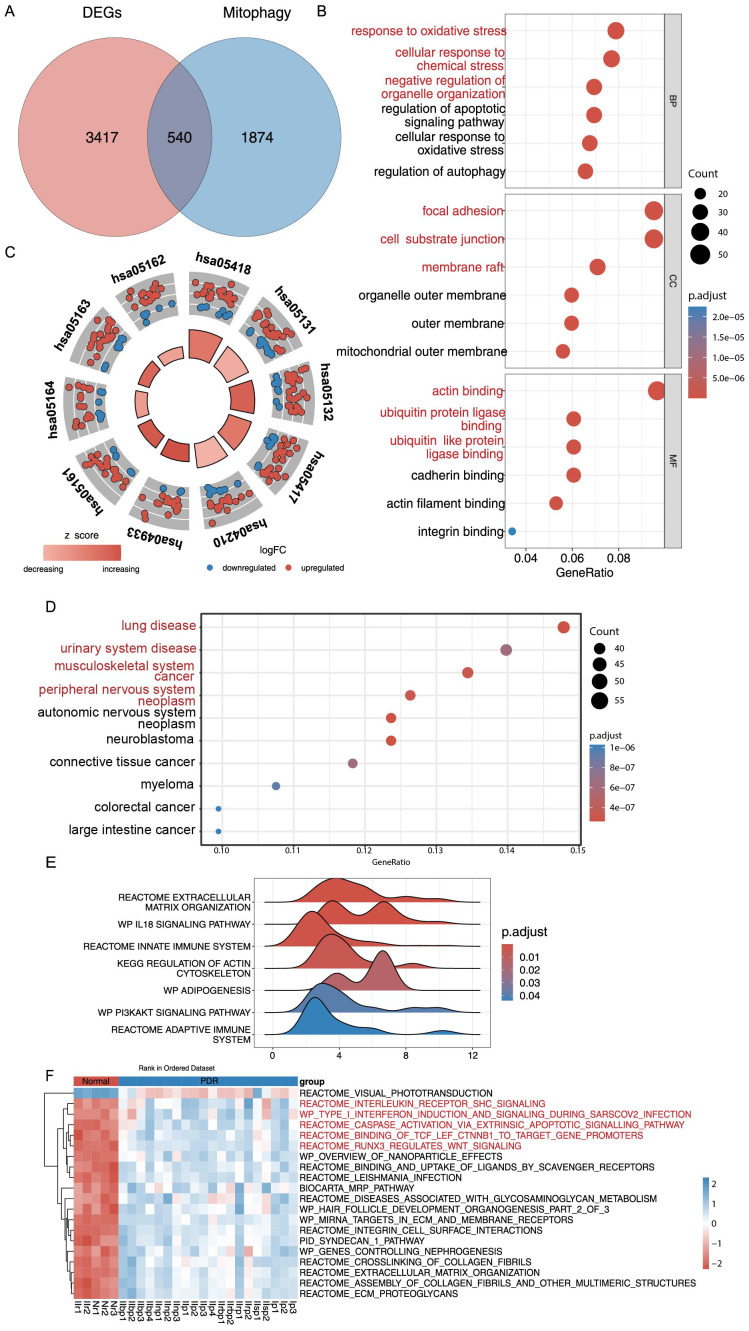
GO, KEGG, DO Enrichment Analysis and GSEA and GSVA analysis of DEMRGs. **(A)** Differentially expressed genes (DEGs) and mitophagy-related genes (MRGs) were taken to intersect to get 540 differentially expressed mitophagy-related genes (DEMRGs). **(B)** DEMRGs enriched in biological process (BP), cellular component (CC), and molecular function (MF) in Gene Ontology (GO) enrichment analysis. **(C)** Kyoto Encyclopedia of Genes and Genomes (KEGG) pathway enrichment analysis of the DEMRGs. **(D)** Disease ontology (DO) enrichment analysis of DEMRGs. **(E)** Enrichment of gene sets with differences in Gene Set Enrichment Analysis (GSEA) analysis. **(F)** Heatmap of specific expression of gene sets with differences in Gene Set Variation Analysis (GSVA) analysis in PDR samples and Control samples.

To our knowledge, mitochondrial malfunction and morphological alterations are linked to DR but remain understudied in PDR. For this reason, genes and pathways relevant to mitophagy in MSigDB and GeneCards were searched for, and 2414 relevant genes were found. After considering the intersection with DEGs, this study identified 540 differentially expressed PDR-associated DEMRGs, and they were targeted for further analysis (**Figure 3A**, **Supplementary Table S4**). This study analyzed 540 DEMRGs using GO, KEGG, and DO enrichment (**Figures 3B–D**, **Supplementary Table S5**). GO analysis showed that DEMRGs were associated with biological processes (e.g., response to oxidative stress, cellular response to chemical stress, and negative regulation of organelle organization), cellular components (e.g., focal adhesion, cell-substrate junctions, and membrane rafts), and other cellular mechanisms (e.g., actin binding, ubiquitin protein ligase binding, and ubiquitin-like protein ligase binding). KEGG analysis showed that the DEMRGs were associated with fluid shear stress, atherosclerosis, lipids, and apoptosis pathways. DO analysis revealed that DEMRGs were linked to various medical conditions and diseases.

GSEA analysis of the DEMRGs revealed that extracellular matrix organization, IL18 signaling pathway, innate immune system, KEGG regulation of the actin cytoskeleton, adipogenesis, PI3K/AKT signaling pathway, adaptive immune system, and other gene sets were significantly enriched in the PDR samples of the Training dataset (**Figure 3E**, **Supplementary Figure S3**, **Supplementary Table S6**). Further analysis of all samples and genes revealed that gene sets such as visual phototransduction were significantly under-enriched in PDR samples. Conversely, interleukin receptor SHC signaling, type I interferon induction and signaling during SARS−CoV−2 infection, caspase activation via extrinsic apoptotic pathways, binding of TCF/LEF ctnnb1 to target gene promoters, and runx3’s regulation of WNT signaling gene sets were significantly and highly enriched in PDR samples (**Figure 3F**, **Supplementary Table S7**).

### 3.4 Constructing a diagnostic model for PDR

**Figure 4:**
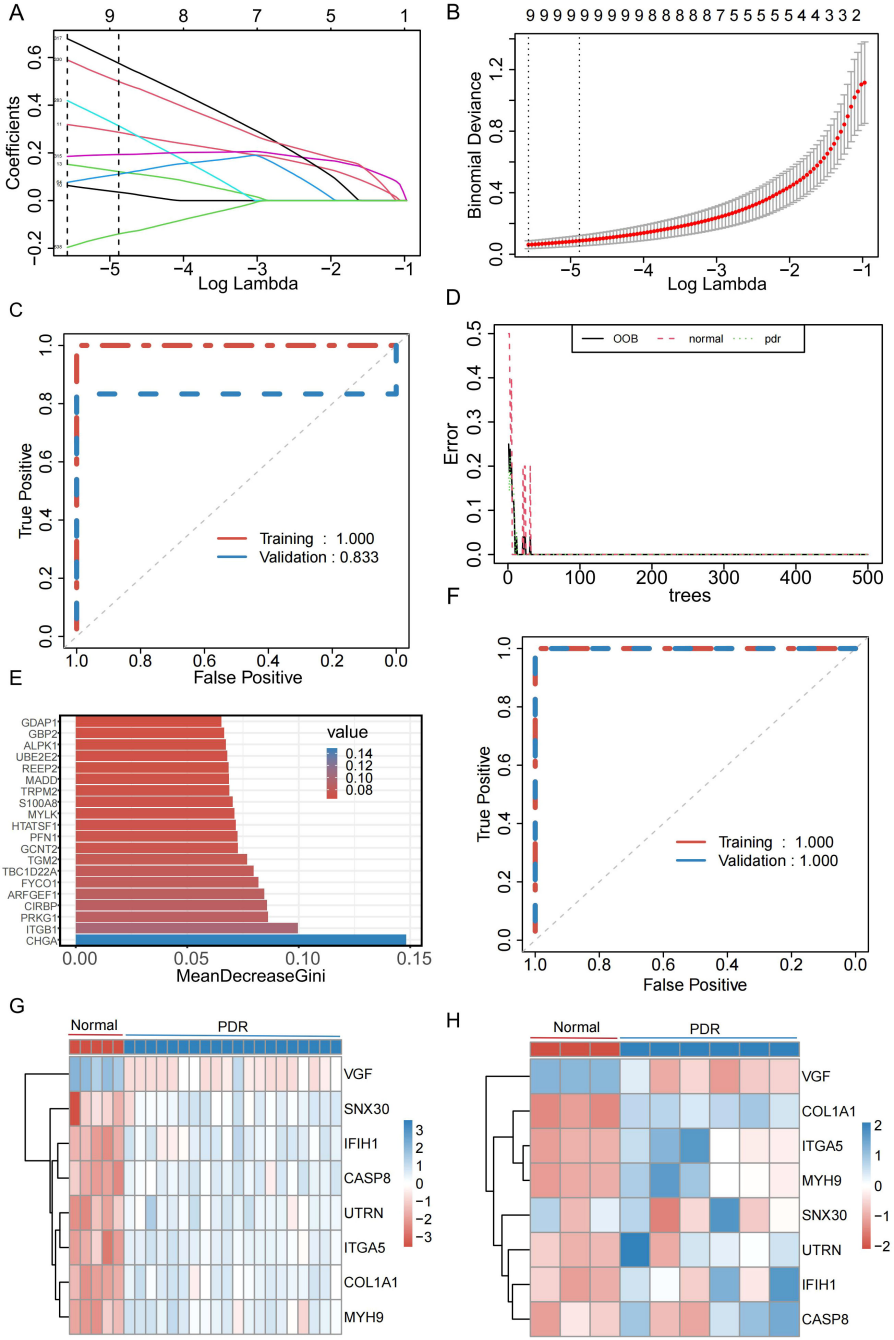
Identification of genes characterized by the PDR model. **(A)** Relationship between Lambada, feature coefficients, and number of features in Least Absolute Shrinkage and Selection Operator (LASSO) regression. **(B)** The best model and the simplest LASSO regression model acquisition. **(C)** Diagnostic efficacy of LASSO regression models in the Training and Validation datasets. **(D)** Changes in error rate with increasing decision trees in random forests. **(E)** Top 20 significant genes in random forest. **(F)** Diagnostic efficacy of random forest models in Training and Validation datasets. **(G)** Expression of model feature genes in the training set. **(H)** Expression of model signature genes in validation set.

To better understand the diagnostic potential of the 540 DEMRGs, a predictive model was constructed to diagnose PDR using the LASSO shrinkage and selection operator, a regression approach to distinguish between patients with PDR and healthy controls. To ensure the reproducibility of the modeling outcomes, a consistent random seed value of 123456 was established. With a gradual increase in lambda, the feature parameters gradually decreased (**Figures 4A, B**). This study used the best model and nine genes were selected (UTRN, COL1A1, MYH9, DOCK8, SNX30, ITGA5, IFIH1, CASP8, and VGF) to model the feature genes for LASSO regression. To assess the robustness of this model, separate calculations were performed for the area under the curve (AUC) in both the Training and Validation datasets. In the Validation dataset, the AUC was recorded at 0.833 (**Figure 4C**), further confirming the model’s efficacy in generalizing its predictive capabilities beyond the training data. To further determine the stability of the model feature genes, a random forest algorithm was used to screen the model feature genes again. The model was stabilized after building 500 decision trees (**Figure 4D**). Simultaneously, the top 200 genes were selected based on their importance as model feature genes of the random forest (**Figure 4E**). To verify the model’s stability, this study calculated the AUC for the Training and Validation datasets. In both datasets, the AUC of the model was 1 (**Figure 4F**). Finally, we took the intersection of the model feature genes obtained from the LASSO and random forest algorithms and identified eight genes (VGF, SNX30, IFIH1, CASP8, UTRN, ITGA5, COL1A1, and MYH9) as model feature mitophagy-related genes in PDR. The expression levels of the eight feature genes in the Training and Validation datasets are shown in **Figures 4G, H**.

### 3.5 Predictive performance and validation of a diagnostic model for PDR

**Figure 5:**
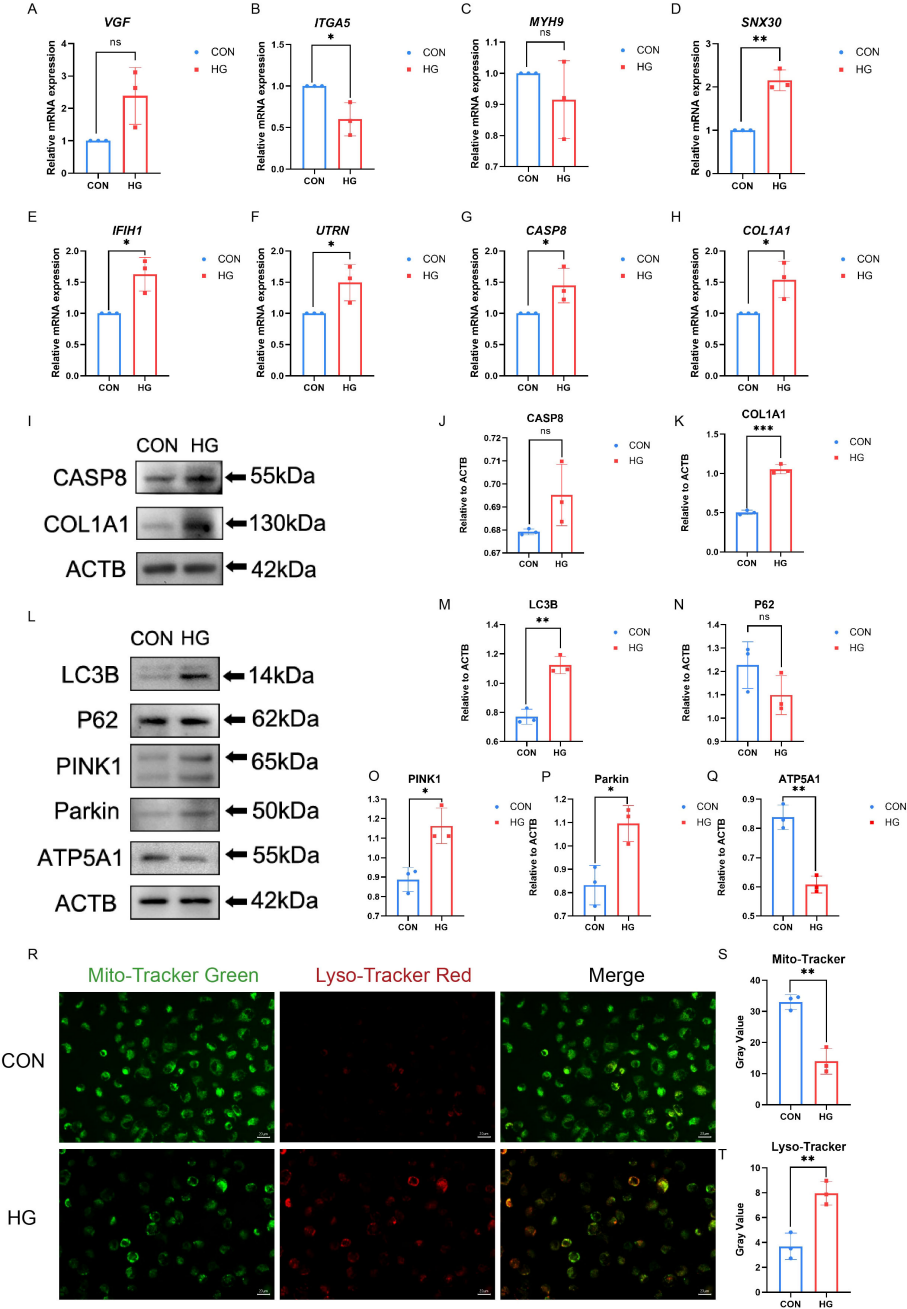
Hub-gene expression and mitophagy markers were measured in ARPE-19 cells. **(A–H)** The mRNA level of VGF, SNX30, IFIH1, CASP8, UTRN, ITGA5, COL1A1, and MYH9 were measured in cell samples by qRT-PCR. P-values were calculated using a two-sided unpaired Student’ s t-test. (*P < 0.05; **P < 0.01; ns, non-significant). **(I–K)** Western blotting analysis of CASP8 and COL1A1 protein expression in ARPE-19 cell samples. P-values were calculated using a two-sided unpaired Student’s t-test. Representative western blotting images and the corresponding statistical analyses are shown (n = 3; *P < 0.05; **P < 0.01; ns, non-significant). **(L–Q)** Western blotting analysis of LC3B, P62, PINK, Parkin and ATP5A1 protein expression in ARPE-19 cell samples. P-values were calculated using a two-sided unpaired Student’s t-test. Representative Western blotting images and the corresponding statistical analyses are shown (n = 3; *P < 0.05; **P < 0.01; ns, non-significant). **(R–T)** The mitochondria and autolysosomes were labeled by Mito- Tracker Green and Lyso-Tracker Red, respectively. Scale bar: 20 μm. Data are presented as the mean ± SD. P-values were calculated using a two-sided unpaired Student’s t-test. (n = 3; *P < 0.05; **P < 0.01; ns, non-significant).

Disrupted mitophagy in RPE cells has been linked to compromised outer blood–retinal barrier in DR. Under high-glucose conditions, RPE cells exhibit dynamic changes in mitophagy-related markers, consistent with perturbations in mitochondrial quality control (37). Owing to RPE cell damage, mitophagy is activated at low glucose levels and inhibited at high glucose levels (21). This study incubated ARPE-19 cells with HG (30mM) at previously reported concentrations to simulate an *in vitro* DR model (38). Interestingly, VGF expression was up-regulated in HG treated ARPE-19 cells compared to that in normal control cells, which was predicted to be down-regulated based on the bioinformatics analysis described above. The expression of ITGA5 was reduced and MYH9 levels remained unchanged in HG treated ARPE-19 cells; these genes were predicted to be upregulated. SNX30, IFIH1, CASP8, UTRN, and COL1A1 expression levels were elevated in ARPE-19 cells under HG conditions, suggesting that these five genes and their downstream signaling pathways may be involved in the progression of PDR by regulating mitophagy in RPE cells (**Figures 5A-H**, **Supplementary Table S8**). Additionally, expression of the two hub genes under HG was examined by western blotting. As shown in **Figures 5I–K**, the exposure of ARPE-19 cells to HG for 24h led to increased COL1A1 protein levels, which was consistent with the trends observed by qRT-PCR, while CASP8 protein levels showed no significant change. To validate these findings, qRT-PCR analysis was performed in iRPE cells previously established in our previous work (15). The results showed that six genes exhibited expression patterns generally consistent with the bioinformatic predictions, with ITGA5 being upregulated compared to ARPE-19 cells (**Supplementary Figure S4**, **Supplementary Table S9**).

To investigate the relationship between high glucose and mitophagy, the changes in LC3B and P62 (31) levels were examined under HG conditions using western blotting, along with the stabilization of PINK1/Parkin (32) and ATP5A1 (33). As shown in **Figures 5L–Q**, HG treatment initially increased the expression of LC3B. P62 expression was slightly reduced, but the change was not statistically significant. ATP5A1 protein levels decreased, indicating reduced inner-mitochondrial membrane content, consistent with ongoing mitochondrial clearance via mitophagy. The mitophagy pathway comprises multiple mechanisms, among which the PINK1/Parkin axis is the most classical and well characterized (34). Western blotting showed that HG stimulation increased PINK1 and Parkin expression in ARPE-19 cells. Furthermore, fluorescence microscopy indicated an increase in mitochondria–lysosome colocalization in ARPE-19 cells after HG stimulation. Following HG exposure, the overlap between Mito-tracker and Lyso-Tracker signals was modestly enhanced, suggesting that mitophagy may be altered under hyperglycemic conditions (**Figures 5R–T**). Additionally, mitochondrial membrane potential (MMP) in ARPE-19 cells under HG stimulation was evaluated using JC-1 staining (**Supplementary Figure S5**) (35). HG increased JC-1 monomer formation and reduced JC-1 aggregate levels. These findings suggest that high glucose may modulate mitophagy and alter the expression of key genes, providing preliminary support for mitophagy dysregulation in PDR and partially addressing the limitations of bioinformatics-based analyses. However, how mitophagy activity changes during the later phases of HG requires further examination.

### 3.6 Prioritization of CASP8 and COL1A1 as hub genes

**Figure 6:**
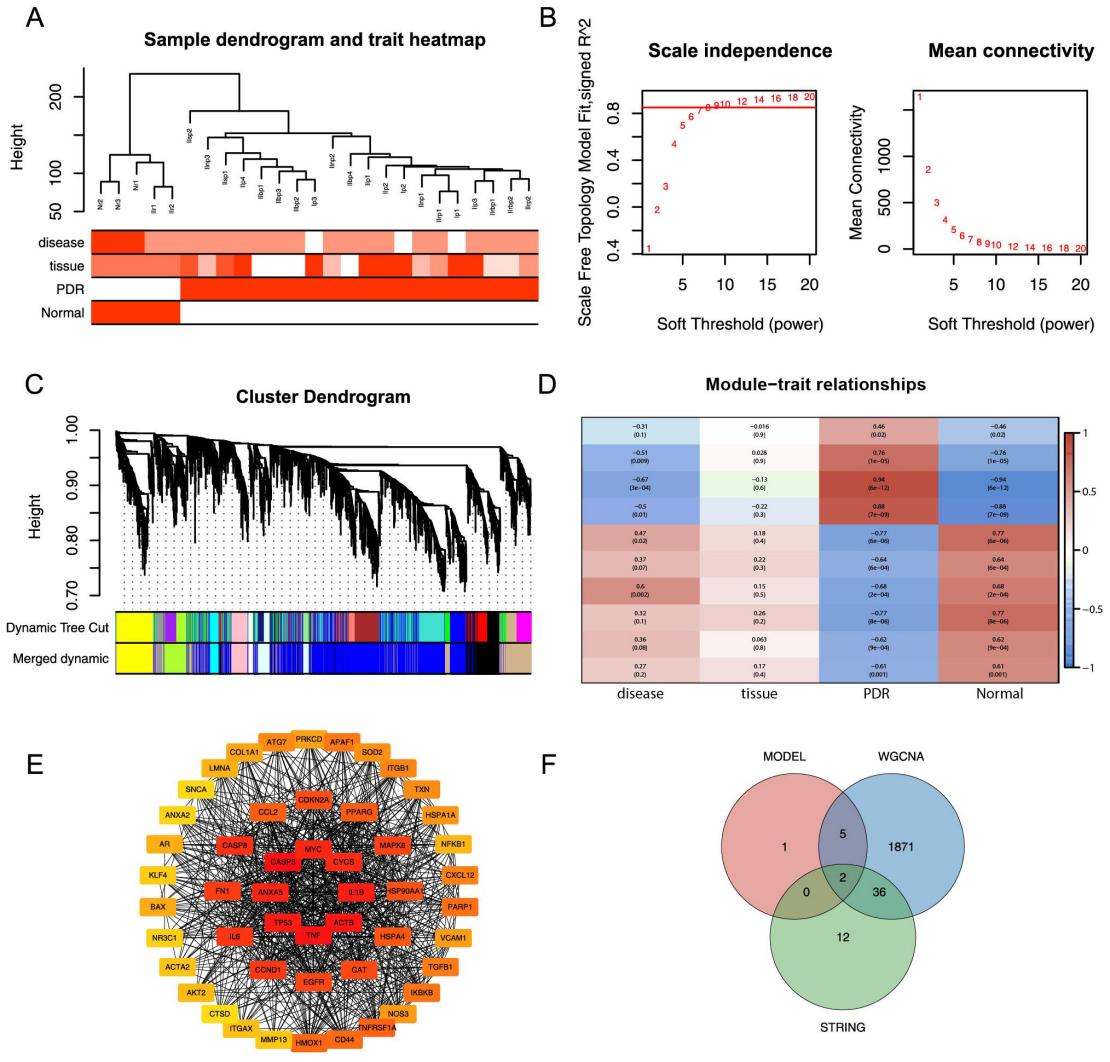
WGCNA analysis and PPI analysis. **(A)** Elimination of outlier samples by cut height. **(B)** Determination of optimal SOFT POWER soft threshold. **(C)** Formation and merging of modules. **(D)** Correlation of module genes with PDR. **(E)** Network graph of the top 50 genes obtained by computing MCC based on cytoHubba. **(F)** Intersection of model feature genes, core module genes, and PPI candidate genes is taken.

In the Training dataset, WGCNA was used to pinpoint gene modules among the DEGs that exhibited the most substantial correlation with PDR. Notably, cluster analysis did not detect any outlier samples, as demonstrated in **Figure 6A**.A soft threshold of 9 was used as the optimal threshold for constructing a scale-free network. The minimum number of genes in the modules was set at 30, resulting in 19 modules formed (**Figure 6B**). Similar modules were merged by setting the minimum distance between the merged modules to 0.2, resulting in 10 modules (**Figure 6C**). After assessing the correlations between various gene modules and clinical characteristics, a correlation heatmap was generated, as represented in **Figure 6D**. This analysis identified the blue module that exhibited the most substantial correlation with PDR. This module encompassed 1914 genes and was subsequently designated as the core module. A PPI network was constructed for DEMRGs using the STRING web tool (**Supplementary Figure S1**). To narrow the focus to the most promising candidates, the maximum clique centrality (MCC) scores were calculated using the CytoHubba plug-in in Cytoscape software. The top 50 genes were selected as potential candidate genes based on pre-scoring, as shown in **Figure 6E**. Finally, eight model feature genes, 1914 blue core module genes, and 50 PPI candidate genes were used as intersections to obtain two hub genes, COL1A1 and CASP8 (**Figure 6F**).

### 3.7 Immunological differences across PDR molecular subtypes

**Figure 7:**
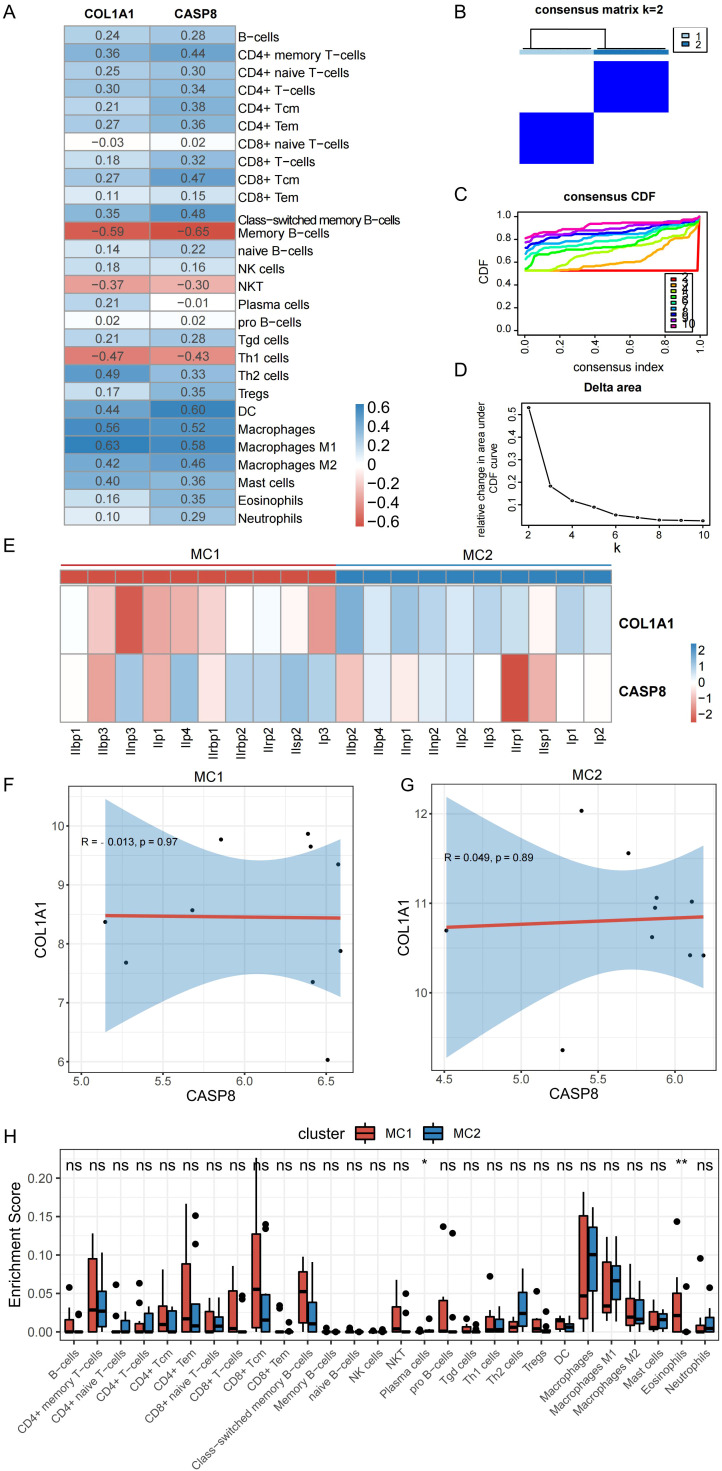
Correlation between hub genes and both the degree of immune cell infiltration and the immune infiltration characteristics of PDR molecular subtypes. **(A)** Correlation between hub genes and degree of immune cell infiltration. **(B)** Clustering heat map at k=2. **(C)** Cumulative distribution curve. **(D)** Area under the cumulative distribution curve. **(E)** Expression of hub genes in different subtypes. **(F, G)** Correlation between CASP8 and COL1A1 in different subtypes. **(H)** Immune cell infiltration of different molecular subtypes of PDR. (*P < 0.05; **P < 0.01; ns, non-significant).

To determine whether the expression levels of mitophagy-related genes were related to immunity, an analysis was conducted to examine the correlation between the expression levels of these hub genes and scores representing immune cell infiltration. As shown in **Figure 7A**, there was some correlation between the two hub genes and most immune cells, with the highest positive correlation between COL1A1 and macrophages (r = 0.63) and the highest negative correlation between COL1A1 and memory B cells (r = –0.59). CASP8 showed the strongest positive correlation with DC (r = 0.60) and the strongest negative correlation with memory B cells (r = –0.65).

To construct PDR molecular subtypes, this study used the expression matrices of the PDR samples in the Training dataset corresponding to the hub genes for consistent clustering. By analyzing the cumulative distribution curves, AUC, and clustered heatmap results (**Figures 7B–D**), k = 2 was chosen as the number of subgroups to classify the PDR samples into two subtypes, classically activated M1 macrophages (MC1) and alternatively activated M2 macrophages (MC2). To explore the distribution of the hub genes in the two isoforms, their expression of hub genes was mapped in different isoform samples (**Figure 7E**). COL1A1 was found to decrease the expression of MC1 isoforms and increase the expression of MC2 isoforms. In the ongoing exploration of the relationship between these two hub genes, their correlations within distinct isoforms were calculated, as illustrated in **Figures 7F, G**. No discernible correlation was observed between these two molecules. This observation strongly implies that these two genes could potentially serve distinct functions, as their isoform-specific correlations were not notably related.

The investigation of the variance in immune cell infiltration between the two subtypes involved a detailed analysis of the disparities in immune cell levels, as illustrated in **Figure 7H**. The results of this analysis showed that the MC1 subtype displayed elevated levels of eosinophils and concurrently lower levels of plasma cells than the other subtype.

## 4 Discussion

PDR is an end-stage and severe type of DR and is an important cause of blindness in patients with diabetes (36). PDR pathogenesis is complex and current research suggests that multiple metabolic pathways are involved in its development, including impaired glutathione metabolism, decreased pantothenic acid and CoA biosynthesis (37), oxidative stress and endoplasmic reticulum stress (38). Abnormalities in these pathways can lead to microvascular complications such as disruption of the blood-retinal barrier, uncontrolled vascular proliferation and neurodegeneration (39). However, the exact pathogenic mechanism of PDR remains understudied. It has been suggested that mitophagy, as the programmed self-degradation of dysfunctional mitochondria, is essential for maintaining cellular homeostasis and cell survival under stress and may be involved in PDR pathogenesis (6). Low glucose (15 mM) induced enhanced mitophagy in RPE cells; however, elevated ROS mediated the inactivation of the key mitophagy proteins, PINK1 and Parkin, and thus inhibited mitophagy in response to HG (50 mM) or hydrogen peroxide stimulation. This suggests that the glucose concentration regulates mitophagy in RPE cells in a dose-dependent manner (6). Kanwar et al. found significantly reduced levels of glutathione, superoxide dismutase (SOD), and other antioxidant molecules in the retinal mitochondria of diabetic mice, which resulted in impaired antioxidant defenses and retinal oxidative stress damage in the RPE (40). These above results suggest that mitophagy is involved in the genesis and development of PDR; however, the specific underlying mechanism remains unclear. Further studies are needed to broaden our understanding of mitophagy in PDR pathogenesis.

This study, we catalogued 540 potential DEMRGs in PDR using bioinformatics analysis, and functional enrichment analysis suggested associations with biological processes such as mitophagy regulation, oxidative stress response, and cellular stress response. Furthermore, this analysis indicated shifts in immune cell composition in PDR, with increased infiltration of CD8+ and CD4+ T cells, DCs, and macrophages, along with a decrease in B cells, NK cells, and regulatory T cells. Next, this study further highlighted eight hub genes associated with PDR using the PPI network and WGCNA analyses, including VGF, SNX30, IFIH1, CASP8, UTRN, ITGA5, COL1A1, and MYH9. The roles of some of these genes in diabetes development and ocular disorders have been extensively studied. For example, ITGA5 promotes angiogenesis in DR through TAK-1/NF-kB activation (41) and COL1A1 may be associated with prefibroblastic cells that cause pre-retinal fibrovascular membranes in patients with PDR (42). However, their role in the regulation of mitophagy in PDR remains unclear.

In the present study, HG-treated ARPE-19 cells were used as a DR model to demonstrate the function of potential mitophagy-related genes. This was because of the following reasons. First, RPE cell dysfunction and loss were identified in a diabetic model. It is associated with macular edema caused by diabetes-induced disruption of the outer blood-retinal barrier. Therefore, RPE cells have been widely used as *in vitro* models for DR studies (43, 44). Second, the RPE contains a high density of mitochondria necessary to fulfill the energy demand; therefore, severe stimulation leads to mitochondrial dysfunction and excessive intracellular ROS production, which further triggers oxidative stress-related mitophagy. This *in vitro* DR model is suitable for studying mitophagy (13). The qRT-PCR results found that five of the eight hub genes prioritized in silico for PDR (SNX30, IFIH1, CASP8, UTRN, COL1A1) showed expression changes consistent with the bioinformatic analysis. The qRT-PCR analysis of iRPE shows that six genes exhibited expression patterns broadly concordant with the bioinformatic predictions, and ITGA5 was upregulated compared with ARPE-19 cells, this may be due to the reinforcement of certain functions during induced differentiation. At the protein level, COL1A1 changes were concordant with the in-silico predictions, supporting its prioritization as a putative target for modulating PDR progression.

Western blotting suggested that mitophagy may be altered under high-glucose stimulation for 24 hours: mitophagy-associated proteins (including LC3 and PINK1/Parkin) tended to increase, whereas the putative inhibitory protein p62/SQSTM1 and the inner mitochondrial membrane complex subunit ATP5A1 showed modest decreases, the findings consistent with an enhancement of mitophagy. In addition, immunofluorescence revealed Mit-Tracker and Lyso-Tracker colocalization, suggesting augmented trafficking of depolarized mitochondria to acidic vesicles, which should be further evaluated using autophagic-flux and lysosomal-function assays. Moreover, high glucose exposure decreased the JC-1 red/green ratio, indicating mitochondrial depolarization and consistent with partial mitochondrial clearance. Taken together, these data are broadly consistent with the *a priori* expectations and provide preliminary experimental support that HG may modulate mitophagy in RPE cells, thereby partially mitigating the limitations of bioinformatics-based inference. However, since the bioinformatics results were derived from the optic disc and the surrounding parts of the retinal tissues of patients with PDR.

Previous studies have reported that IFIH1 is involved in mitophagy through the RIG-I/MDA5-MAVS pathway (45) and UTRN deficiency impairs cellular mitochondrial quality control (46), suggesting that CASP8 is a dysregulated gene involved in mitophagy in human periodontal ligament stem cells (47). The decreased in COL1A1 protein levels is associated with the loss of mitophagy and insufficient collagen secretion (48). CASP8 and COL1A1 are closely related to mitophagy, and CASP8 and COL1A1 were also designated as hub genes by CytoHubba analysis. The relationship between these genes and mitophagy has only been partially reported, and further investigation is required to elucidate the mechanisms by which mitophagy is regulated in PDR models and cells.

However, because this analysis is based on bulk retinal transcriptomic data that aggregate heterogeneous tissue compartments and cell types, the interpretation of differentially expressed genes and pathway signals may be affected to some extent. In addition, pathway and gene-set inferences depend on continually updated databases, in which redundancy and overlapping annotations can blur specificity. Accordingly, this study is framed as a systems level exploration of molecular features, aiming to identify common signals shared across compartments rather than those confined to a single tissue. Future work will incorporate rigorously compartment stratified samples and apply single cell RNA sequencing and spatial transcriptomics to validate expression and functional differences that are specific to compartments and cell types. Moreover, among the eight prioritized candidates, only five showed concordant changes, which may reflect biological differences between complex patient tissues and a single retinal pigment epithelium cell line. Validation in additional human retina-derived cell lines will be required to substantiate the predictive and regulatory roles of these mitophagy-related genes in PDR.

## 5 Conclusions

To sum up, we prioritized eight candidate mitophagy-related genes associated with PDR, among which CASP8 and COL1A1 emerged as putative hub genes. The findings suggest that SNX30, IFIH1, CASP8, UTRN, and COL1A1 may influence the onset and progression of PDR by modulating mitophagy. Nevertheless, these conclusions are hypothesis-generating, as they are derived primarily from public datasets and algorithmic inference, their relevance to disease biology requires further confirmation in animal models and patient-derived tissues. Future work should integrate functional genetics and protein-level assays to delineate the regulatory mechanisms of these key genes comprehensively and to establish the reliability and translational value of these candidates as clinical biomarkers or therapeutic targets.

## Data Availability

The original contributions presented in the study are included in the article/**Supplementary Material**, further inquiries can be directed to the corresponding author/s.

## Glossary

DR: Diabetic retinopathy
PDR: Proliferative diabetic retinopathy
AIF: apoptosis-inducing factor
AMD: age-related macular degeneration
ROS: excessive reactive oxygen species
RPE: retinal pigment epithelium
DEGs: differentially expressed genes
MRGs: mitophagy-related genes
FVMs: fibrovascular membranes
GO: Gene Ontology
DEMRGs: Differentially expressed mitophagy-related genes
CPM: counts per million
BP: biological processes
MF: molecular functions
CC: cellular components
DO: Disease ontology
GSEA: Gene Set Enrichment Analysis
GSVA: Gene Set Variation Analysis
LASSO: Least Absolute Shrinkage and Selection Operator
STRING: Search Tool for the Retrieval of Interacting Genes/Proteins
MCC: Maximal Clique Centrality
PAM: partitioning around medoids
hUCMSCs: human umbilical cord mesenchymal stem cells
iRPE: induced retinal pigment epithelium
HG: high glucose
NC: normal control
SG: standard glucose
DCs: Dendritic cells
MC1: classically activated M1 macrophage
MC2: alternatively activated M2 macrophage
PCA: Principal Component Analysis.
