# Supplementary material for: Identification and validation of key mitophagy-related biomarkers in the pathogenesis of proliferative diabetic retinopathy
Source: Front Endocrinol (Lausanne). 2025 Oct 29;16:1652898. doi: 10.3389/fendo.2025.1652898 (PMC12605334; doi:10.3389/fendo.2025.1652898)
Supplement: Supplementary file 1 [file DataSheet1.docx]

Supplementary Material

# Supplementary Figures and Tables

## Supplementary Figures

**
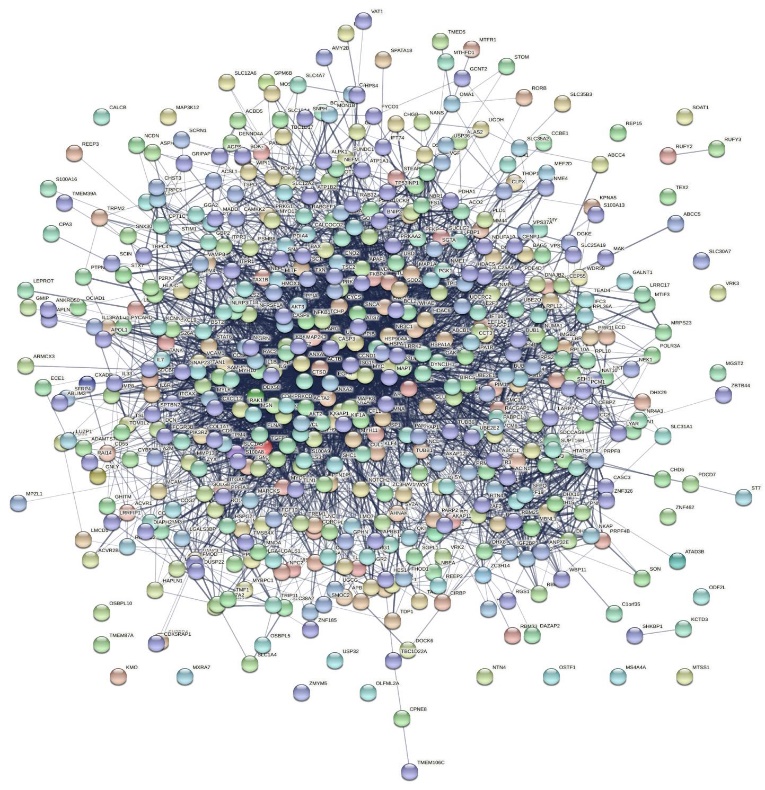
**

**Supplementary Figure 1.** PPI network of DEMRGs.

**
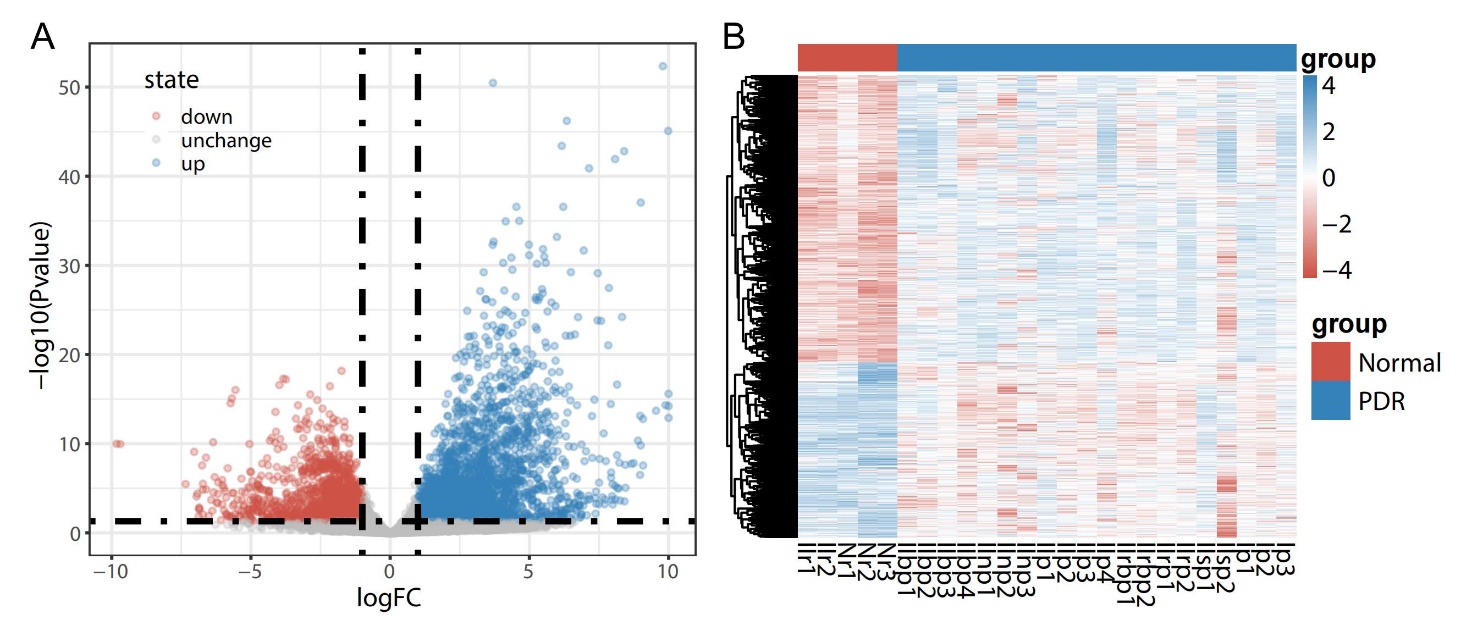
**

**Supplementary Figure 2.** Analysis of DEGs. (A) Volcano plot showing the results of differential analysis, 2460 genes with up-regulated expression and 1497 genes with down-regulated expression (red represents down-regulated expression and blue represents up-regulated expression). (B) Heatmap showing the expression of DEGs between the PDR samples and Control samples (red represents down-regulated expression and blue represents up-regulated expression).


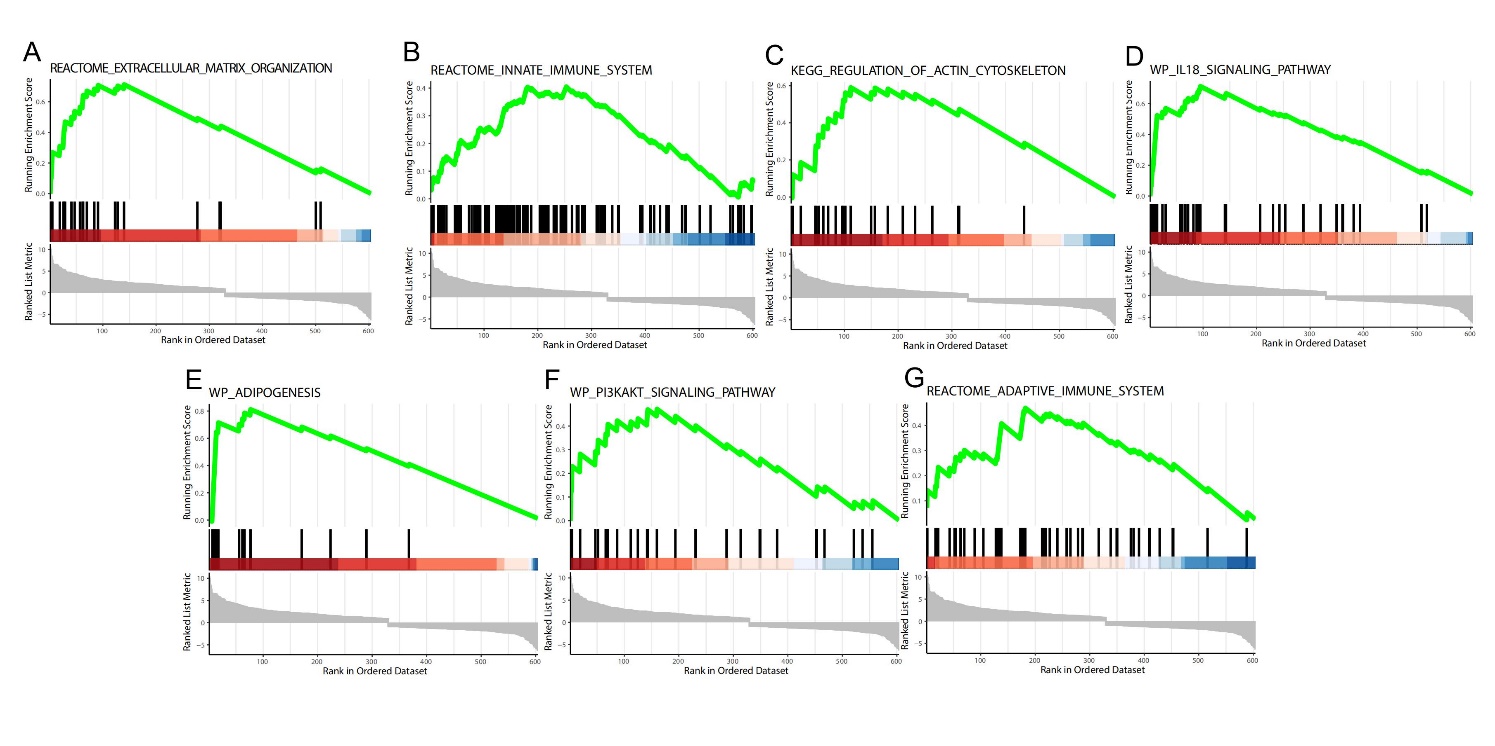


**Supplementary Figure 3.** GSEA analysis of DEMRGs. (A-G) Enrichment of gene sets with differences in GSEA analysis.


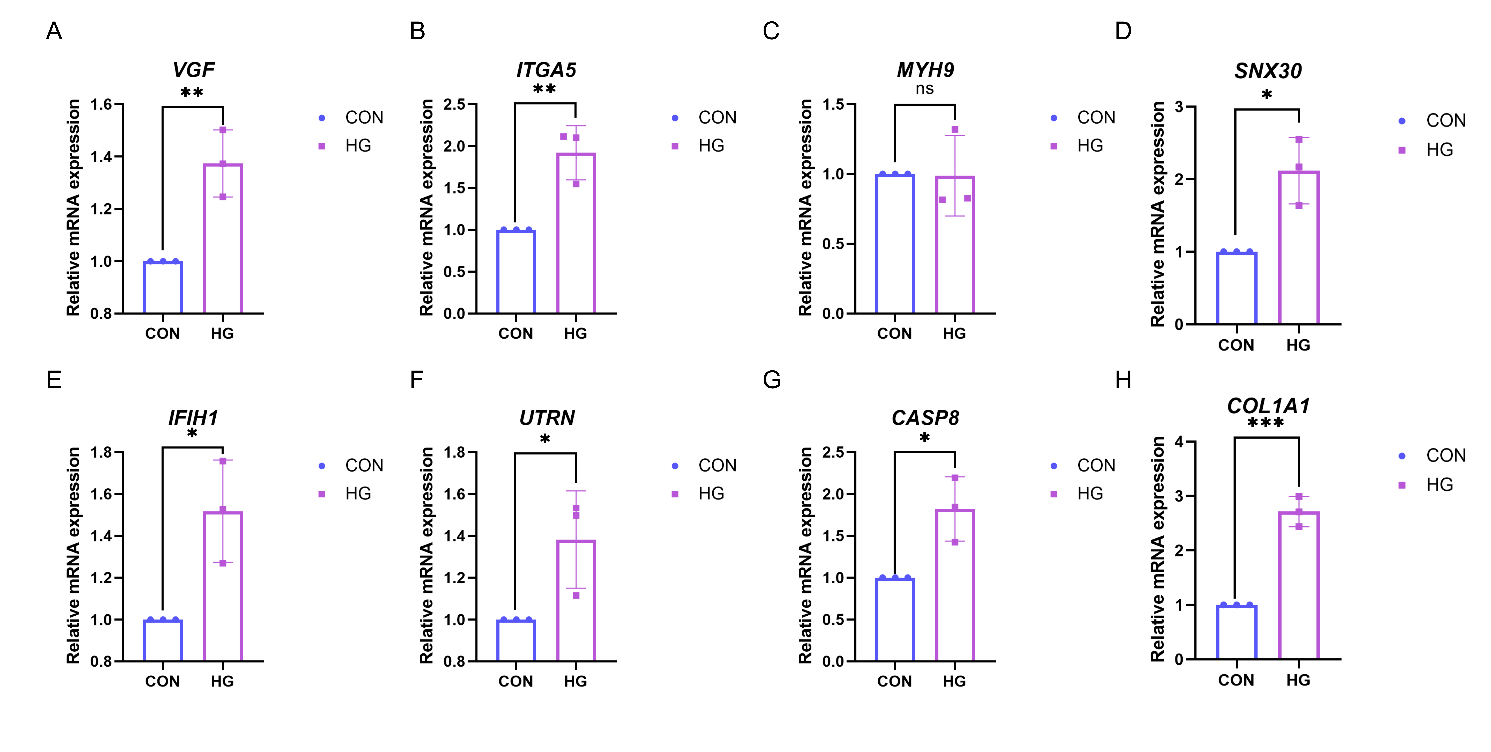


**Supplementary Figure 4.** The mRNA level of 8 hub genes was measured in iRPE cells.

(A-H) The mRNA level of VGF, SNX30, IFIH1, CASP8, UTRN, ITGA5, COL1A1, and MYH9 were measured in cell samples by qRT-PCR. P-values were calculated using a two-sided unpaired Student’s t-test. (n=3; *P < 0.05; **P < 0.01; ns, non-significant).


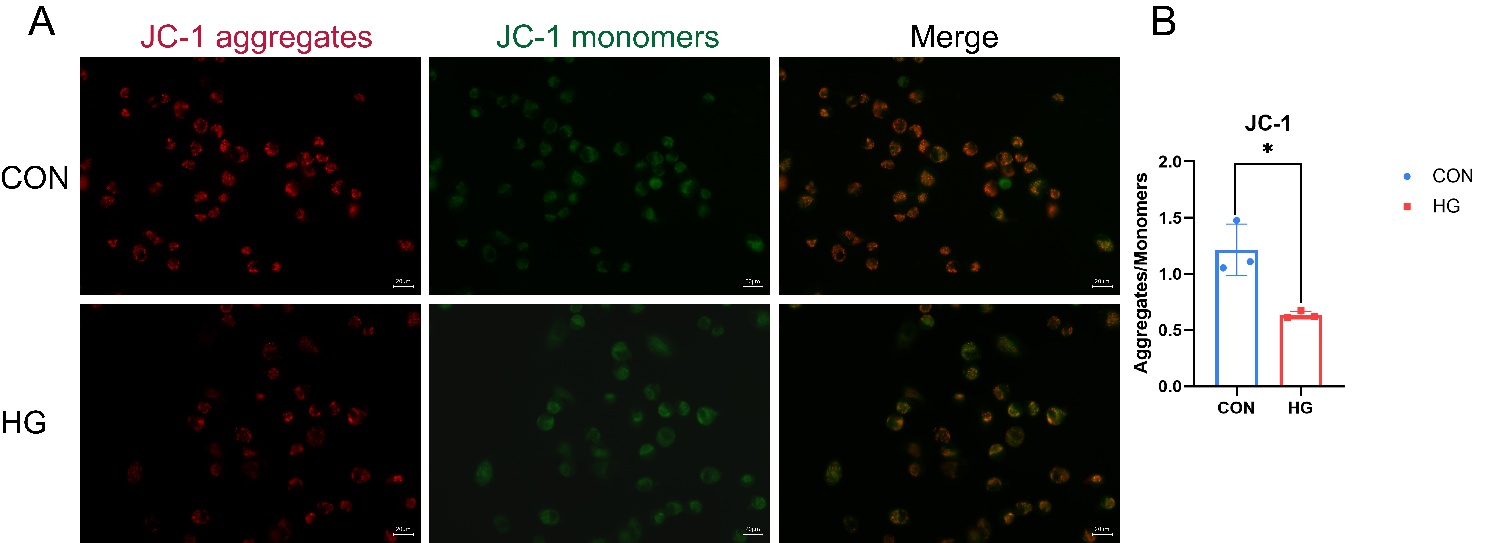


**Supplementary Figure 5.** Mitochondrial membrane potential (MMP) was determined by JC-1 staining. The ratio of aggregates/monomers was compared in the right bar graph. Scale bars, 20 μm. Data are presented as the mean ± SD. P-values were calculated using a two-sided unpaired Student’s t-test. (n = 3; *P < 0.05; **P < 0.01; ns, non-significant).

## Supplementary Tables

**Supplementary Table 1.** Mitophagy-Related Genes.

**Supplementary Table 2.** Immune Cell Enrichment Level.

**Supplementary Table 3.** Antibodies for Western Blotting.

**Supplementary Table 4.** Differential Mitophagy- Related Genes.

**Supplementary Table 5.** GO/KEGG/DO Enrichment Results.

**Supplementary Table 6.** GSEA Enrichment Results.

**Supplementary Table 7.** Pathway Enrichment.

**Supplementary Table 8.** ARPE-19 cells qRT-PCR Data Representative of Three Independent Experiment.

**Supplementary Table 9.** iRPE cells qRT-PCR Data Representative of Three Independent Experiment.
